# Molecularly engineering a dual-drug nanoassembly for self-sensitized photodynamic therapy via thioredoxin impairment and glutathione depletion

**DOI:** 10.1080/10717544.2022.2141920

**Published:** 2022-11-09

**Authors:** Hongyuan Zhang, Zhiqiang Kong, Ziyue Wang, Yao Chen, Shenwu Zhang, Cong Luo

**Affiliations:** Department of Pharmaceutics, Wuya College of Innovation, Shenyang Pharmaceutical University, Shenyang, P.R. China

**Keywords:** Photodynamic therapy, dual-drug nanoassembly, self-sensitized, thioredoxin impairment, glutathione depletion

## Abstract

Photodynamic therapy (PDT) has been extensively investigated as a spatiotemporally noninvasive and controllable modality for cancer treatment. However, the intracellular antioxidant systems mainly consisting of thioredoxin (Trx) and glutathione (GSH) significantly counteract and prevent reactive oxygen species (ROS) accumulation, resulting in a serious loss of PDT efficiency. To address this challenge, we propose that PDT can be improved by precisely blocking antioxidant systems. After molecular engineering and synergistic cytotoxic optimization, a DSPE-PEG_2K_-modified dual-drug nanoassembly (PPa@GA/DSPE-PEG_2K_ NPs) of pyropheophorbide a (PPa) and gambogic acid (GA) is successfully constructed. Interestingly, GA can effectively destroy intracellular antioxidant systems by simultaneously inhibiting Trx and GSH. Under laser irradiation, the cell-killing effects of PPa is significantly enhanced by GA-induced inhibition of the antioxidant systems. As expected, PPa@GA/DSPE-PEG_2K_ nanoparticles demonstrate potent antitumor activity in a 4T1 breast tumor-bearing BALB/c mouse xenograft model. Such a carrier-free self-sensitized nanotherapeutic offers a novel co-delivery strategy for effective PDT.

## Introduction

Malignant tumors continue to be a principal cause of death, disability, and socioeconomic disruption worldwide (Yu et al., 2020; Ding et al., 2022; Jana & Zhao, 2022; Jiang et al., 2022; Y. Liu, Zhang et al., 2022). Although chemotherapy is the mainstay of clinical cancer treatment, drug resistance and severe side effects often occur, resulting in unsatisfactory clinical treatment results (Weir et al., 2011; Zhang et al., 2021b). To deal with disordered and intractable tumors, novel treatment modalities such as photodynamic therapy (PDT) have been widely investigated (Jia et al., 2021; Zhang et al., 2021). Different from other treatments, PDT as a local treatment modality has attracted considerable attention due to its high selectivity and low toxicity (Dai et al., 2019). Thus, various photosensitizers (PSs) have been designed to perform effective antitumor activity by converting the near-infrared light into tumor-localized reactive oxygen species (ROS) (Xie et al., 2021; Liu et al., 2021; Yang et al., 2022). In other words, PDT has become an up-and-coming option for therapeutic regimens for cancer.

Despite unique advantages, PDT efficiency is often limited by the hostile tumor microenvironment, especially the intracellular antioxidant systems (Zhou et al., 2016; Yang et al., 2021). There are two independent antioxidant systems in the body: the thioredoxin (Trx) system [including NADPH, thioredoxin reductase (TrxR) and Trx] and the glutathione (GSH) system [including NADPH, GSH, and GSH reductase (GR)] (Song et al., 2012; Zhou et al., 2016). In these two systems, GR and TrxR catalyze the transfer of electrons from NADPH to GSSG and Trx, respectively. Moreover, such two systems could participate in various subsequent reactions to maintain redox balance, and essential detoxification mechanisms to protect tumor cells from damage by ROS (Liang et al., 2018). The inhibition of the antioxidant system in tumor cells during PDT can significantly improve the efficacy of PDT (Liu et al., 2017). Therefore, high-efficiency and synchronous delivery of antioxidant blockers and PSs to the tumor is a reliable option for enhanced PDT, but it is also a significant challenge.

With the rapid development of nanotechnology and biomedicine, various nanocarriers have been designed to deliver anticancer drugs (Yang et al., 2020; Liu et al., 2021; J. Yu, Chu et al., 2021; Liu et al., 2022; Shan et al., 2022; Zhang S, Wang et al., 2022). Although some drugs can be successfully delivered to the tumor site depending on the encapsulation of carriers, some apparent disadvantages of these nanoparticles (NPs) constructed based on vehicle materials are exposed simultaneously, such as premature drug leakage, low drug loading, and vector-related toxicity (Chen et al., 2022; Wang et al., 2022). Recently, carrier-free nanodrug delivery system (CFNDS) has gradually come to the attention of researchers (Zhang et al., 2020; Zhang et al., 2021a). In the CFNDS, the drugs can form stable nanoassemblies without the assistance of any carrier, which can be the self-assembly of a single drug or the co-assembly of multiple drugs (Zhang et al., 2020, Zhang S, Sun et al., 2022). Since no carrier material is involved in the assembly process, the drug loading efficiency and delivery efficiency are greatly improved, and the system toxicity induced by the carrier is also avoided (Zhang et al., 2020). The emergence and development of CFNDS have brought excellent prospects and principles for drug delivery to tumor tissues.

**Figure 1.**
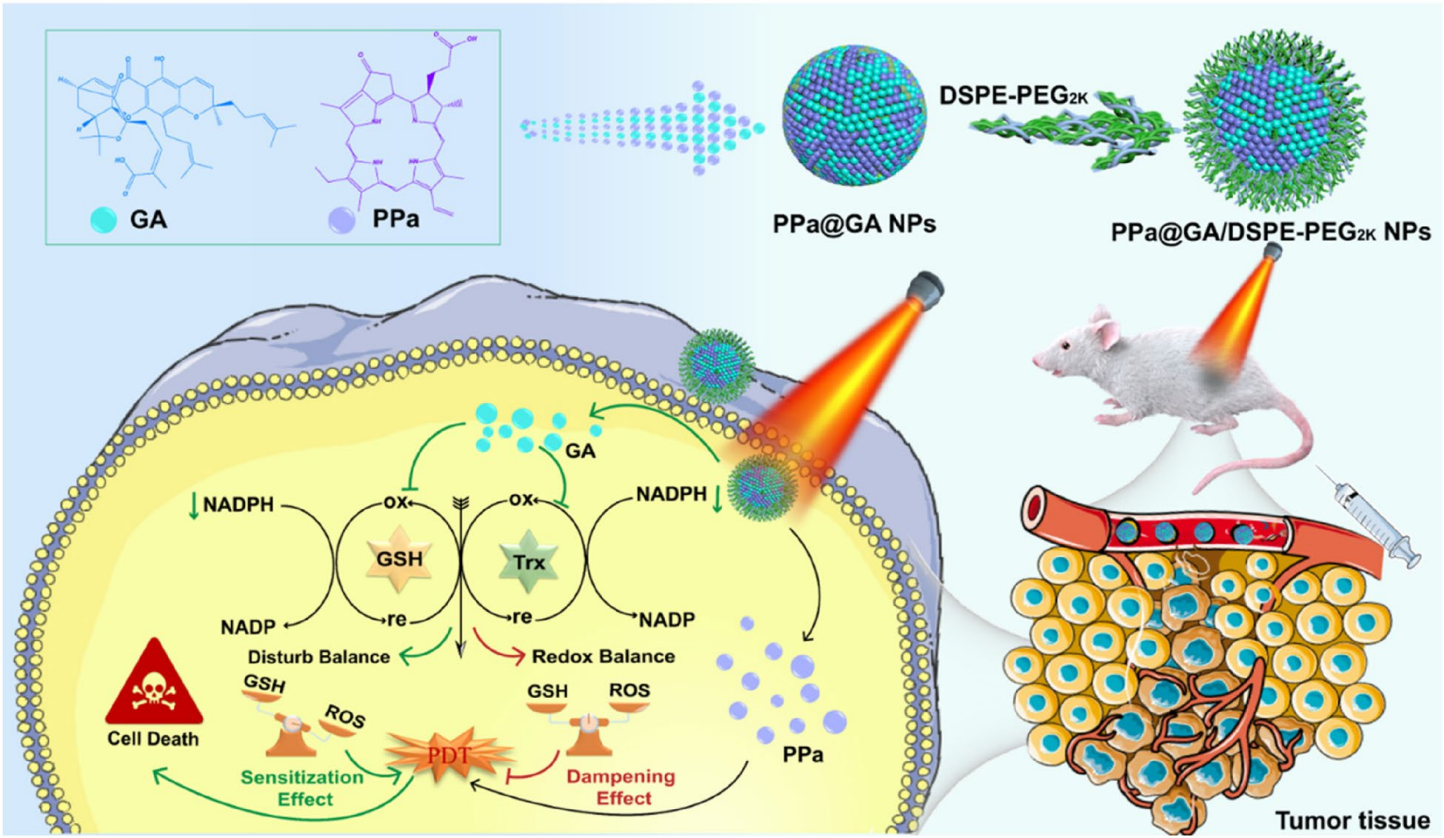
Schematic illustration of the tumor-specific sensitized PDT with an antioxidation-inhibited strategy for enhancing anti-tumor efficacy.

Based on the aforementioned problems and challenges, we herein constructed a dual-drug carrier-free nanoassembly to enhance the tumor’s PDT efficacy (Figure 1). In this assembly process, pyropheophorbide a (PPa) and gambogic acid (GA) can easily co-assemble into uniform NPs without other carriers or excipients. Afterward, non-PEGylated NPs (PPa@GA NPs) were prepared by the one-step nanoprecipitation method. To improve the stability of PPa@GA NPs, DSPE-PEG_2K_ (20 wt%) was utilized as a stabilization modifier to construct the PEGylated NPs (PPa@GA/DSPE-PEG_2K_ NPs). Once PPa@GA/DSPE-PEG_2K_ NPs entered the tumor cells, GA could directly damage the antioxidant system in the cells by inhibiting Trx and GSH. As a result, GA could significantly improve the effect of PDT, and performed a significant synergistic anti-tumor effect with PPa under laser irradiation. Really as expected, PPa@GA/DSPE-PEG_2K_ NPs exerted a highly effective tumor suppressor effect in a 4T1 breast tumor-bearing BALB/c mice. This dual-drug delivery strategy provides a promising nanoplatform and referable regimen for sensitizing PDT or other combination therapies.

## Materials and methods

### Materials

PPa was obtained from Shanghai Xianhui Pharmaceutical Co., Ltd. DSPE-PEG_2K_ was purchased from A.V.T. (Shanghai) Pharmaceutical Co., Ltd. GA was obtained from Dalian Meilun Biotech Co., Ltd., China. 2’,7’-dichlorofluorescin diacetate (DCFH-DA) was purchased from Genview. GSH assay kit was obtained from Nanjing Jiancheng Bioengineering Institute. Hoechst 33342 was purchased from BD Biosciences, USA. Mouse TXN ELISA Kit was purchased by Shanghai COIBO Biotechnology Co., Ltd. Cell culture dishes and plates were bought from NEST Biotechnology Co., Ltd (Wuxi, China). Other solvents were of analytical or high performance liquid chromatography (HPLC) grade.

### Screening the optimal synergistic dose ratio of GA and PPa

The synergistic cytotoxicity of PPa and GA were evaluated by 3-(4,5-dimethyl-2-thiazolyl)-2,5-diphenyl-2-H-tetrazolium bromide (MTT) assay in 4T1 cells. In brief, 4T1 cells (2 × 10^3^/well) were seeded into 96-well plates and cultured at 37 °C and 5% CO_2_ for 12 h. Then, the cells were treated with various concentrations of PPa solution, GA solution, or the mixture of PPa and GA at a wide range of molar ratios [molar ratios (PPa/GA) of 5:1, 4:1, 3:1, 2:1, 1:1, 1:2, 1:3, 1:4, and 1:5]. The cells were continued to incubate into the incubator. After 4 h of incubation, all groups (except GA solution groups) were exposed to the laser (660 nM, 50 mW/cm^2^) for 5 min. The cells were incubated for 44 h under the same condition. Then, 25 μL of MTT solution was added and incubated for 4 h at 37 °C. The mixture was discarded and 200 μL of dimethyl sulfoxide (DMSO) was added to dissolve the formazan. Finally, the ultraviolet (UV) absorbance was measured by Varioskan lux multimode microplate reader (Thermo Scientific, USA).

The synergistic effect of PPa and GA was evaluated by calculating combination index (CI) using the following formula:

$$\text{CI}=\;A\;\times \;\text{PPa}@{\text{GA}}_{\text{combine}/}{\text{GA}}_{\text{alone}}+\;B\;\times \;\text{PPa}@{\text{GA}}_{\text{combine}}/{\text{PPa}}_{\text{alone}}$$

where *A* is the dose proportion of GA in the PPa/GA mixture; *B* is the dose proportion of PPa in the PPa/GA mixture; GA_alone_ was utilized to calculate the IC_50_ when the cells received GA only; PPa@GA_combine_ was utilized to calculate the IC_50_ when GA was combined with PPa; PPa_alone_ was utilized to calculate the IC_50_ when the cells received PPa only. The results were divided into additivity (CI = 1), synergistic effect (CI < 1), and antagonistic effect (CI > 1).

### Preparation and characterization of nanoassemblies

A one-step nanoprecipitation method was utilized to prepare the dual-drug nanoassemblies. Briefly, PPa (2.0 mg) and GA (2.0 mg) were dissolved in tetrahydrofuran (1 mL), respectively. Then, the mixed solution containing PPa solution (144 μL) and GA solution (56 μL) was added dropwise to deionized water (2 mL) with stirring (800 rpm, 4 min) to obtain PPa@GA NPs. The DSPE-PEG_2k_ (20 wt%) was slowly added into PPa@GA NPs to obtain PPa@GA/DSPE-PEG_2K_ NPs. Finally, the organic solvent in the formulation was removed by rotary evaporation (30 °C). The size and zeta potential of the nanoassemblies were measured by Zetasizer (NanoZS, Malvern Co., UK). The morphologies of these nanoassemblies were observed by transmission electron microscopy (TEM, JEOL 100CX II, Japan).

The loading rate was calculated by the following equation (a–c):

(a) $$\text{PPa}\;\left(\%\right)\;=\;m\text{PPa}/\left(m\text{PPa}\;+\;m\text{GA}\;+\;m\text{DSPE}-{\text{PEG}}_{2K}\right)\;\times \;100$$

(b) $$\text{GA}\;\left(\%\right)\;=\;m\text{GA}/\left(m\text{PPa}\;+\;m\text{GA}\;+\;m\text{DSPE}-{\text{PEG}}_{2K}\right)\;\times \;100$$

(c) $$\text{Total}\;\left(\%\right)\;=\;\left(m\text{PPa}\;+\;m\text{GA}\right)/\left(m\text{PPa}\;+\;m\text{GA}\;+\;m\text{DSPE}-{\text{PEG}}_{2K}\right)\;\times \;100$$

where *m*PPa represents the total mass of PPa in the formulation; *m*GA represents the total mass of GA in the formulation; *m*DSPE-PEG_2K_ represents the total mass of *m*DSPE-PEG_2K_ in the formulation.

### Colloidal stability

The colloidal stability of nanoassemblies was detected by the changing of particle size. First, to investigate the effect of PEG amount on stability, we detected the particle size changes of NPs with different DSPE-PEG_2K_ content in PBS by Zetasizer. Then, to further investigate the colloidal stability of PPa@GA NPs and PPa@GA/DSPE-PEG_2K_ NPs were incubated in PBS containing 10% FBS on a shaker (37 °C) for 24 h, respectively. The particle sizes of the NPs were measured using Zetasizer at preset time points (0, 1, 2, 4, 8, 12, and 24 h). Moreover, to investigate the colloidal stability of nanoassemblies under acidic conditions, the particle sizes of the NPs incubation with PBS (pH 7.4) were measured using Zetasizer at preset time points (0, 1, 2, 4, 8, 12, and 24 h).

### Ultraviolet and fluorescence spectra

The ultraviolet and fluorescence spectra of PPa solution, PPa@GA NPs, and PPa@GA/DSPE-PEG_2K_ NPs with/without laser irradiation (5 min, 660 nm, 50 mW/cm^2^) at an equivalent PPa concentration of 10 μg/mL were obtained on a varioskan lux multimode microplate reader (Thermo Scientific, USA).

### Co-assembly mechanisms

The co-assembly mechanisms of PPa and GA were explored via molecular docking experiment (Zhang et al., 2021). The Autodock Vina software was utilized to stimulate the process of molecular docking. First, the 3D structures of PPa and GA were constructed with energy minimization in MMFF94 force field. Then, the molecular docking calculations of PPa–GA, PPa–PPa, and GA–GA were conducted using the Vina protocol in Yinfo Cloud Platform. After internal clustering, semi-flexible docking with maximum pose output was carried out and the docking process was accomplished. After that, the detailed assembly mechanisms of nanoassembly were verified by adding intermolecular interaction breakers (sodium dodecyl sulfate (SDS), urea, and NaCl) (Wang et al., 2020; Zhang et al., 2021). The particle size of NPs after 8-h incubation with SDS, urea, and NaCl was measured using Zetasizer.

### *In vitro* singlet oxygen (^1^O_2_) generation

The *in vitro* ^1^O_2_ production capacity of nanoassemblies was evaluated using the SOSG kit. PPa solution, GA solution, PPa@GA solution, PPa@GA NPs, and PPa@GA/DSPE-PEG_2K_ NPs (2 μM, PPa equivalent) were mixed with SOSG (2 μM) and incubated in deionized water (1 mL). The generated ^1^O_2_ from formulations with/without laser (5 min, 660 nm, 50 mW/cm^2^) was measured using a microplate reader.

### Cell culture

4T1 was gained from American Type Culture Collection (ATCC). 4T1 cells were cultured in RPMI 1640 medium containing 10% FBS, penicillin (100 units/mL), and streptomycin (100 μg/mL). The cell was incubated in a humidified atmosphere (5% CO_2_) at 37 °C.

### Cellular uptake

Cellular uptake of NPs was investigated by analyzing the intracellular fluorescence intensity of PPa. Briefly, 4T1 cells were seeded in the 24-well plates with cell crawling at a density of 1 × 10^5^ cells per well and cultured for 12 h. Then the cultured media were removed and the cells were incubated with PPa solution, PPa@GA NPs and PPa@GA/DSPE-PEG_2K_ NPs for 2 and 4 h (200 nM, PPa equivalent). After incubation, the cells were washed thrice with cold PBS and fixed with paraformaldehyde (4%). Finally, Hoechst 33342 was used to stain the cell nucleus for 10 min. The intracellular fluorescence signals were imaged by confocal laser scanning microscopy (CLSM, C2, Nikon, Japan). Quantitative analysis of intracellular fluorescence intensity was investigated by flow cytometry.

### *In vitro* cytotoxicity of nanoassmblies

MTT assay was performed to assess the cytotoxicity of nanoassemblies. Briefly, 4T1 cells (2 × 10^3^/well) were seeded into a 96-well plate and incubated for 12 h. Then, the old medium was replaced with the fresh media containing gradient concentrations of GA solution, PPa solution, PPa@GA solution, PPa@GA NPs, and PPa@GA/DSPE-PEG_2K_ NPs (the last four groups were all set to light and non-light groups), respectively. After incubation for 4 h, the light groups were irradiated with a laser (660 nm, 50 mW/cm^2^) for 5 min and were further incubated for 44 h. Finally, MTT assay was performed to assess the cell viability after treatments.

### Intracellular ROS and GSH assay

The intracellular ROS and GSH levels were measured using DCFH-DA and GSH assay kits. To assess the intracellular ROS level, 4T1 cells (1 × 10^5^/well) were seeded into 24-well plates and incubated for 12 h at 37 °C. Then, old media were replaced with fresh culture media containing PPa solution, GA solution, PPa@GA solution, PPa@GA NPs, PPa@GA/DSPE-PEG_2K_ NPs at a PPa dose equivalent of 200 nM and incubated for 4 h (37 °C, dark). Afterward, all groups were washed with the cold PBS, treated with DCFH-DA (10 μM), and incubated for 30 min (37 °C). Then, the cells of laser-treat groups were exposed to the laser light (660 nm, 50 mW/cm^2^) for 5 min. Finally, the cells were washed with cold PBS three times, and the cellular fluorescence signals were recorded using an Eclipse Ti-U inverted microscope (Nikon Corp., Tokyo, Japan). For GSH assay, the process of cell culture and drug treatment was the same as ROS assessment, and a microplate reader performed the next procedure following the manufacturer’s instruction.

### Thioredoxin activity

Inhibition effect of Trx activity by GA was evaluated using TXN ELISA Kit. Briefly, 4T1 cells (6 × 10^5/^well) were seeded into Petri dishes (100 mm) and incubated for 12 h. Then, the old media were replaced with fresh medium containing GA solution (67 nM), PPa solution (200 nM), PPa@GA solution, PPa@GA NPs, or PPa@GA/DSPE-PEG_2K_ NPs (200 nM, PPa equivalent) and incubated for 4 h. Then, the cells of laser-treat groups were exposed to the laser light (660 nm, 50 mW/cm^2^) for 5 min. After next incubation for 44 h, the cells were washed three times with the cold PBS and centrifuged. To detect the components in the cells, the cell suspension was diluted with PBS (pH 7.4), and the cell concentration reached about 1 × 10^6^ cells/mL. Repeated freezing and thawing were done to destroy cells and release their contents. Then, the mixture was centrifugated for about 20 min (3000 rpm) and the supernatant was carefully collected. The next procedures were performed following the manufacturer’s instruction. The absorbance was measured at 450 nm with a microplate reader, and the content of Trx was calculated by standard curve.

### Animal studies

Sprague-Dawley (SD) rats and BALB/c mice were utilized according to the requirements and regulations of the Animal Ethics Committee of Shenyang Pharmaceutical University. In animal experiments, PPa solution, GA solution, and PPa@GA solution were prepared by the similar method. First, PPa, GA, or PPa@GA powder were added to a mixed solution of absolute ethyl alcohol and cremophor EL (1/1, vol/vol). After 3 min of vortex, the mixed solution was diluted by PBS for intravenous administration.

### Pharmacokinetics study

SD rats (200–220 g) were used to study the pharmacokinetic profiles. PPa solution, PPa@GA NPs, and PPa@GA/DSPE-PEG_2K_ NPs (2.5 mg/kg, PPa equivalent) were injected intravenously into the male rats, respectively (*n* = 3). At specified time intervals (0.033, 0.083, 0.25, 0.5, 1, 2, 4, 8, 12, and 24 h), about 300 μL of blood from the ophthalmic venous plexus of each rat was removed and centrifuged (8 × 10^3^ rpm, 5 min) to obtain the supernatant. The concentrations of PPa in the plasma were determined by a microplate reader (Thermo Scientific, USA).

### *Ex vivo* biodistribution

The *ex vivo* biodistribution was investigated in 4T1 tumor-bearing mice. The female BALB/c mice were inoculated with 4T1 cells (5 × 10^6^ cells) on the right flank of each mouse. When the tumor volume reached about 300 mm^3^, the mice were administered intravenously with PPa solution, PPa@GA NPs, and PPa@GA/DSPE-PEG_2K_ NPs at a PPa dose equivalent of 2.5 mg/kg. After administration for 2, 4, 8, and 12 h, the mice were dissected to obtain the major organs and tumors for ex *vivo* imaging by in vivo imaging system (IVIS® Lumina LT Series III, PerkinElmer) (*n* = 3).

### *In vivo* synergetic antitumor efficacy and safety

The *in vivo* antitumor efficacy was evaluated in 4T1 tumor-bearing mice. Briefly, the 4T1 cells were subcutaneously inoculated into the right flank of each female BALB/c mouse. When the tumor volume reached 150 mm^3^, mice were randomized into eight groups (*n* = 6): PBS, GA solution, PPa solution + L, PPa@GA NPs, PPa@GA/DSPE-PEG_2K_ NPs, PPa@GA solution + L, PPa@GA NPs + L, and PPa@GA/DSPE-PEG_2K_ NPs + L. Various formulations were injected intravenously into the 4T1 tumor-bearing female BALB/c mice at a PPa dose of 3 mg/kg at intervals of 2 days for a total of five times. After injection for 4 h, the tumor of mice in laser-irradiated groups were exposed to laser (5 min, 660 nm, 50 mW/cm^2^). The body weight and tumor volume of mice were monitored every day. After two days of the last treatment, the mice were sacrificed to obtain the major organs (heart, liver, spleen, lung, kidney) and tumors for hematoxylin-eosin (H&E) staining. Moreover, tumor tissues were used for histological evaluation with TdT-mediated dUTP nick end labeling (TUNEL) staining and the collected plasma was utilized for hepatorenal function analysis.

### Statistical analysis

All the data were calculated using GraphPad Prism 8 and presented as mean ± standard deviation (SD). The significant differences between groups were identified by *t* test or one-way analysis of variance (ANOVA), and *p* < .05 was considered as statistically significant.

## Results and discussion

### Screening the synergistic dose ratio of PPa and GA

As we mentioned, PPa and GA can be easily co-assembled to form NPs without any excipients. To determine the optimal synergistic ratio, we performed *in vitro* synergistic antitumor evaluation of PPa and GA at a wide range of molar ratios (5:1, 4:1, 3:1, 2:1, 1:1, 1:2, 1:3, 1:4, and 1:5). As shown in Supplemental Figure S1 and Tables S1 and S2, the optimized molar ratio of 3:1 (PPa/GA) exhibited the most excellent cytotoxicity with the synergy index was 0.52 in 4T1 cell. Comprehensively considering its synergistic antitumor efficiency and formulation characterization (Supplemental Table S3), we finally selected the drug ratio as 3:1 (PPa/GA) and the following relevant studies were carried out using the ratio.

### Preparation and characterization of nanoassemblies

**Figure 2.**
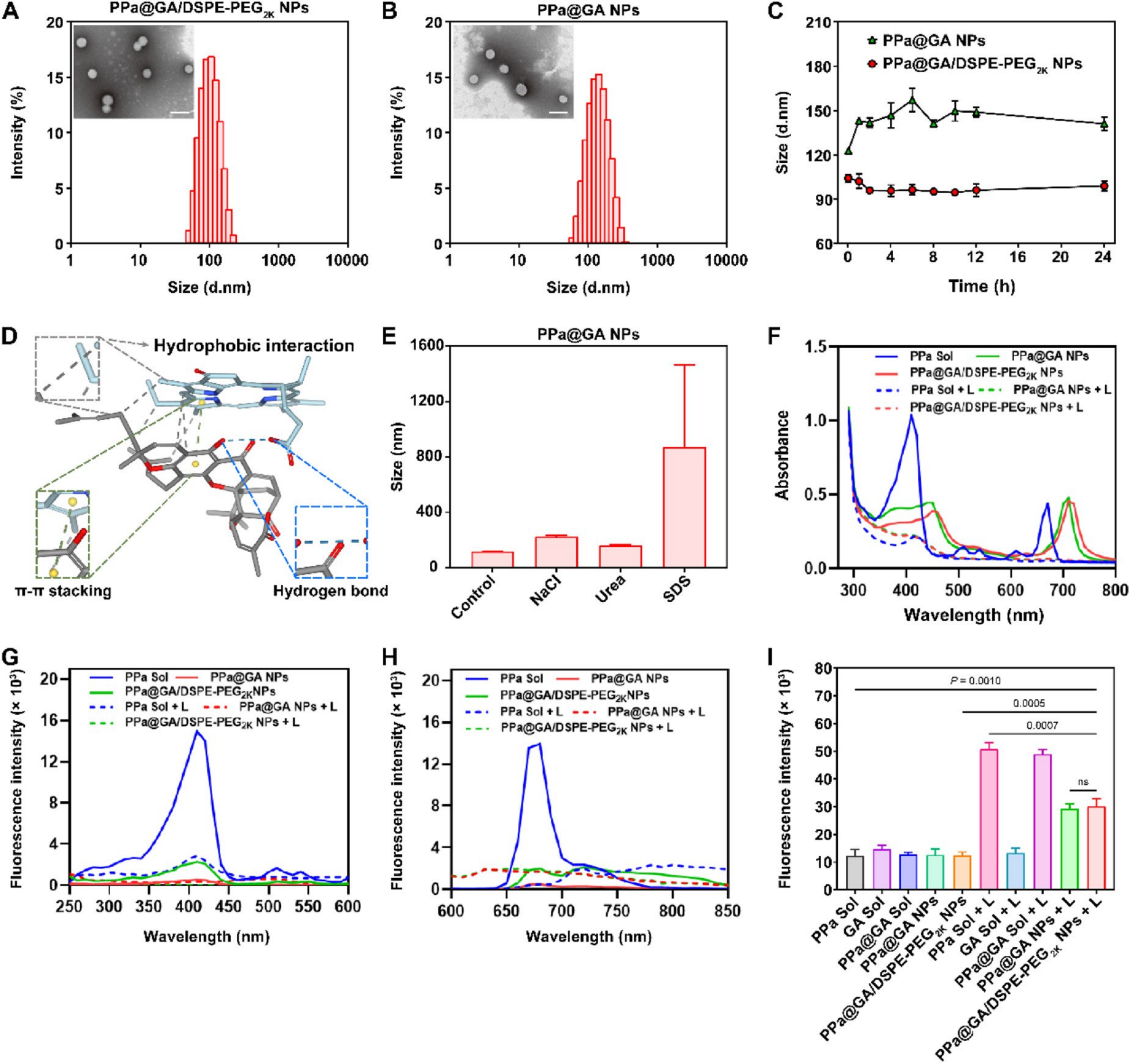
Characterization of PPa@GA/DSPE-PEG_2K_ NPs. A and B: Intensity size distribution profiles and TEM image of PPa@GA/DSPE-PEG_2K_ NPs (A) and PPa@GA NPs (B). Scale bar, 200 nm. C: Colloidal stability of PPa@GA NPs and PPa@GA/DSPE-PEG_2K_ NPs in the PBS (7.4) containing 10% FBS. D: Molecular docking simulation of PPa and GA. E: The particle size changes of NPs before and after incubation with NaCl, urea, and SDS (10 mM) for 8 h. F: UV absorption spectra at 300–800 nm. G and H: The PPa fluorescence spectra from 250 nm to 600 nm (G) and 600–850 nm (H) of PPa sol, PPa@GA NPs and PPa@GA/DSPE-PEG_2K_ NPs. I: The properties of singlet oxygen generation at an equivalent PPa dose of 2 μM with/without laser irradiation (660 nm, 50 mW/cm^2^, 5 min).

Non-PEGylated NPs (PPa@GA NPs) were prepared by one-step nanoprecipitation method. Generally, PEGylated NPs was able to maintain better colloidal stability compared with non-PEGylated NPs. To resist salting out of non-PEGylated NPs and improve the stability of PPa@GA NPs, DSPE-PEG_2K_ (20, wt%) was used as a stabilization modifier to form PPa@GA/DSPE-PEG_2K_ NPs (Supplemental Figure S2). As shown in Figure 2(A,B) and Supplemental Table S4, the particle sizes of PPa@GA NPs and PPa@GA/DSPE-PEG_2K_ NPs were about 125 nm and 95 nm, respectively, and the zeta potentials were approximately –20 mV and –30 mV, respectively. As expected, PEGylation decoration significantly improved the colloidal stability of PPa@GA/DSPE-PEG_2K_ NPs. As exhibited in Figure 2(C), the particle size of PPa@GA/DSPE-PEG_2K_ NPs almost did not change significantly during the incubation with PBS medium (pH 7.4) containing 10% FBS for 24 h, while a significant increase in particle size could be observed in PPa@GA NPs under the same conditions. Moreover, PPa@GA/DSPE-PEG_2K_ NPs revealed good colloidal stability under acidic conditions (PBS, pH 6.5) (Supplemental Figure S3). These results demonstrated that PPa@GA/DSPE-PEG_2K_ NPs had better colloid stability than PPa@GA NPs, which was beneficial for drug delivery *in vivo.*

**Figure 3.**
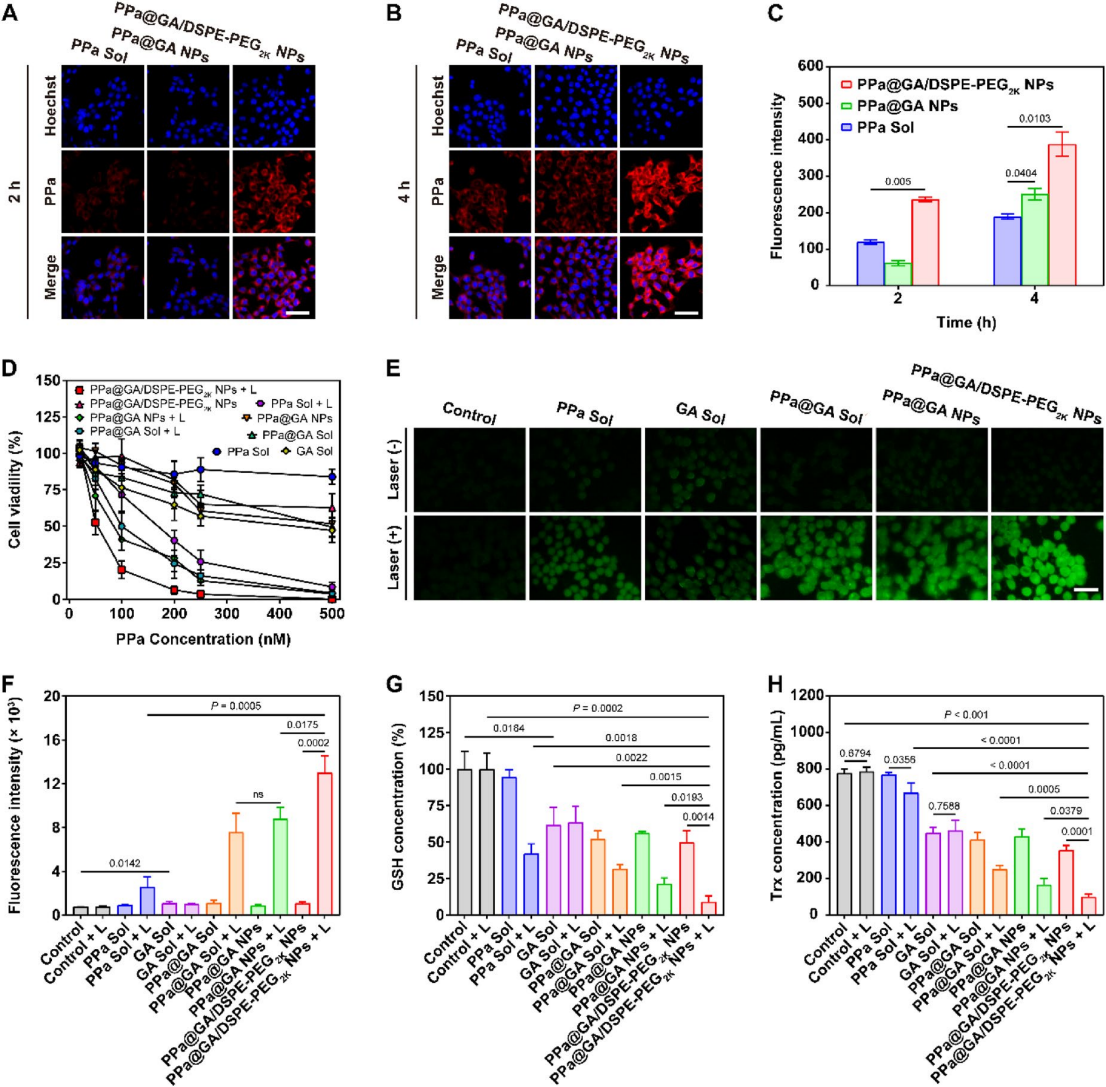
The cellular uptake and combination effects of PPa@GA/DSPE-PEG_2K_ NPs on generating ROS, depleting GSH and inhibiting thioredoxin 1 protein expression *in vitro*. A and B: Confocal microscopic images of the cellular uptake of 4T1 cells after treating PPa sol, PPa@GA NPs, and PPa@GA/DSPE-PEG_2K_ NPs, at a PPa equivalent dose of 200 nM for 2 h and 4 h. Scale bar, 10 μm. C: Quantitative analysis of cellular uptake in 2 h and 4 h from flow cytometry. D: Synergistic cytotoxicity against 4T1 cells with/without laser irradiation (660 nm, 50 mW/cm^2^, 5 min). E: Cellular ROS generation of 4T1 cells after different treatments for 4 h with/without laser irradiation (660 nm, 50 mW/cm^2^, 5 min) by inverted microscope. Scale bar, 2 μm. F: Quantitative analysis of cellular ROS generation in 4 h from flow cytometry. G: Relative percentages of GSH expression after different treatments for 4 h. H: Cellular Trx concentration of 4T1 cells after different treatments for 48 h.

### Co-assembly mechanism exploration

The assembly mechanism of PPa and GA was investigated by molecular docking simulation technique and intermolecular force destruction. As illustrated in Figure 2(D), multiple intermolecular forces of PPa and GA were involved in the process of nanoassembly, including hydrophobic interaction, π–π stacking interaction, and hydrogen bond. In addition to between PPa and GA molecules, the same intermolecular PPa–PPa and GA–GA also had assembly driving forces (Supplemental Figure S4). All the affinities of GA–PPa, GA–GA, and PPa–PPa were less than –4 kcal/mol, suggesting good binding capacity between molecules (Supplemental Table S5).

**Figure 4.**
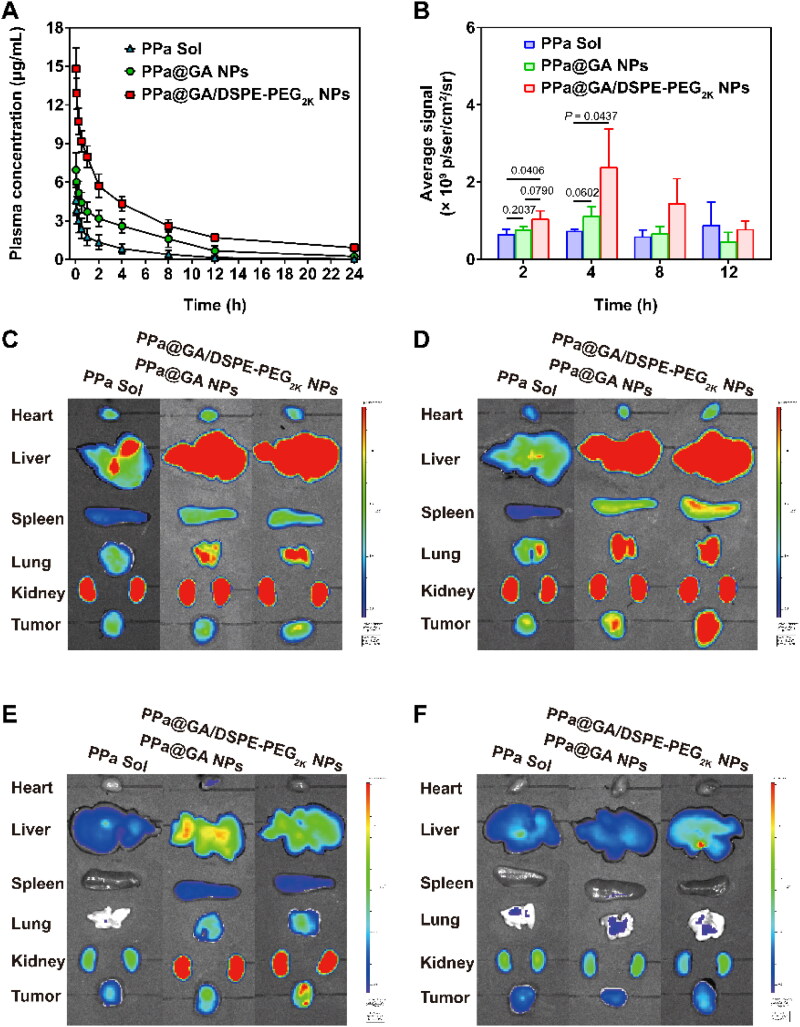
The pharmacokinetic profiles and *ex vivo* biodistribution of dual-drug nanoassemblies. A: The concentration-time curves of PPa Sol, PPa@GA NPs, and PPa@GA/DSPE-PEG_2K_ NPs at a PPa equivalent dose of 2.5 mg/kg (*n* = 6). B: Quantitative analysis of tumors at 2, 4, 8, and 12 h. C–F: The fluorescent imaging of major organs and tumors at 2 h (C), 4 h (D), 8 h (E), and 12 h (F).

Moreover, to further investigate the intermolecular forces, SDS, urea, and NaCl were used to destroy hydrophobic interaction, hydrogen bond, and electrostatic interaction. As shown in Figure 2(E), the particle size of PPa@GA NPs incubated in SDS solution increased dramatically, indicating that hydrophobic interaction contributed to the assembly process. By contrast, slight particle size variation was observed in PPa@GA NPs incubated with urea and NaCl solution.

As illustrated in Figure 2(F), the UV absorption spectra showed that the absorption spectra of PPa@GA NPs and PPa@GA/DSPE-PEG_2K_ NPs were significantly red-shifted compared with that of PPa solution, confirming the existence of π–π stacking interaction in the nanoassemblies (Wang et al., 2020). Furthermore, fluorescence spectra of PPa were explored before and after co-assembly with GA. As shown in Figure 2(G,H), the fluorescence intensities of the excitation and emission spectra of PPa@GA NPs and PPa@GA/DSPE-PEG_2K_ NPs significantly decreased, which could be ascribed to the aggregation caused quench (ACQ) effect and suggested the successful formation of the nanoassemblies (Zhanget al., 2021). Moreover, we further detected the effect of PDT on nanoassemblies. After laser irradiation, the redshift UV absorption peak of the nanoassembly disappeared, and the UV absorption peak of PPa Sol also decreased significantly. Similarly, after laser irradiation, the fluorescence intensities of PPa Sol, PPa@GA NPs, and PPa@GA/DSPE-PEG_2K_ NPs also dramatically declined. The decrease of UV absorption and fluorescence intensity indicated that the structures of nanoassemblies were destroyed and intermolecular forces disappeared due to the photobleaching of PPa (Zhang et al., 2021). Together, these results demonstrated that a variety of forces, mainly hydrophobic, participated in the assembly process of PPA and GA, which promoted the successful formation of nanoassemblies. Moreover, such an assembly behavior of PPa and GA might be destroyed by laser irradiation due to PPa photobleaching.

### *In vitro*^1^O_2_ generation capacity

We then explored the *in vitro* ^1^O_2_ generation capacity of nanoassemblies with/no laser irradiation using the SOSG probe. As expected, the PPa-contained laser irradiated groups revealed higher ^1^O_2_ generation capacity than those without laser treatment, suggesting the PPa-driven and laser-sensitized ^1^O_2_ generation capacity. Moreover, compared with PPa solution and PPa@GA solution, the amount of ^1^O_2_ generated from PPa@GA NPs and PPa@GA/DSPE-PEG_2K_ NPs exhibited a specific decrease, which could be ascribed to the ACQ effect (Figure 2I).

### Cellular uptake

The cellular uptake efficiency of drugs was intuitively correlated with the final therapeutic efficiency. The cellular uptake efficiency of nanoassemblies was investigated by virtue of the fluorescence of PPa. As shown in Figure 3(A–C), PPa solution, PPa@GA NPs, and PPa@GA/DSPE-PEG_2K_ NPs were internalized into 4T1 cells in a time-dependent manner. Notably, the cellular uptake of PPa solution and nanoassemblies at 4 h was significantly higher than that at 2 h. Compared with PPa solution, the fluorescence intensities of PPa@GA NPs and PPa@GA/DSPE-PEG_2K_ NPs were higher at both 2 h and 4 h. Moreover, PPa@GA/DSPE-PEG_2K_ NPs exhibited higher cellular internalization efficiency than PPa@GA NPs. We speculated that non-PEGylated PPa@GA NPs had lower stability in the cellular medium, resulting in reduced cellular uptake efficiency.

### Insight into synergetic antitumor mechanism

The favorable colloidal stability, ^1^O_2_ generation, and cellular uptake capacity of PPa@GA/DSPE-PEG_2K_ NPs motivated us to study the synergistic cytotoxicity further. The synergistic antitumor effect of nanoassemblies was investigated using MTT assay. As shown in Figure 3(D) and Supplemental Table S6, there was almost no toxicity in the PPa solution without the laser-treated group. Under laser irradiation (660 nm, 50 mW/cm,^2^ 5 min), the combined group, including PPa@GA solution, PPa@GA NPs, and PPa@GA/DSPE-PEG_2K_ NPs, exhibited more cytotoxicity than single PPa solution or GA solution. Compared with PPa@GA solution and PPa@GA NPs, PPa@GA/DSPE-PEG_2K_ NPs exerted a more potent antitumor activity, which could be ascribed to the rational dose ratio, great colloidal stability, and cellular uptake of the PEGylated nanoassembly. To explore the synergetic antitumor mechanism, we first examined the intracellular ROS generation ability of nanoassemblies after various treatments. As depicted in Figure 3(E,F), GA-media groups showed the ability to increase intracellular ROS in addition to PPa laser exposure groups, suggesting the capacity to break the redox balance. Compared with PPa or GA alone, a higher ROS level was observed in the cells treated with PPa@GA, PPa@GA NPs, and PPa@GA/DSPE-PEG_2K_ NPs due to the synergistic effect of PPa and GA. As expected, PPa@GA/DSPE-PEG_2K_ NPs demonstrated the highest ROS accumulation in accordance with the cytotoxicity results.

Moreover, we further investigated the reason for ROS elevation, intracellular GSH content and Trx expression were assessed by GSH kit and TXN ELISA Kit, respectively (Figure 3(G,H)). Under laser irradiation (660 nm, 50 mW/cm,^2^ 5 min), ROS produced by PPa could counteract and decrease the level of GSH. Importantly, GA could synergistically downregulate the expression of GSH with PPa under laser irradiation, which was beneficial to increasing ROS accumulation and sensitized PDT. Meanwhile, GA was also capable of inhibiting Trx expression, performing a synergistic downregulation of Trx with PPa under laser irradiation. These results demonstrated that GA could reduce GSH and Trx levels to inhibit the intracellular antioxidant system, synergistically amplifying the antitumor activity of PDT.

### Pharmacokinetics and biodistribution

The pharmacokinetic profiles of PPa@GA solution and PPa@GA NPs, PPa@GA/DSPE-PEG_2K_ NPs were investigated in SD rats. As illustrated in Figure 4(A) and Supplemental Table S7, PPa could be quickly cleared from the blood with a short half-life. Compared with PPa solution, PPa nanoassemblies significantly extended blood circulation time and half-life. Moreover, PPa@GA/DSPE-PEG_2K_ NPs exhibited better pharmacokinetic behavior than PPa@GA NPs due to the higher colloidal stability. The *C*_max_ and area under concentration–time curve (AUC) of PPa@GA/DSPE-PEG_2K_ NPs were about two times higher than those of PPa@GA NPs.

The enhanced long circulation of nanoassemblies could certainly promote tumor-specific accumulation. To test this conjecture, we further the *ex vivo* biodistribution of nanoassemblies in 4T1 tumor-bearing mouse model. As depicted in Figure 4(B–F) and Supplemental Figure S5, the PPa@GA solution and PPa@GA NPs, PPa@GA/DSPE-PEG2K NPs were successfully accumulated into tumors. Such an accumulation presented a rise and then decline trend from 2 h to 12 h, and the peak drug accumulation in tumors was found at around 4 h post-injection for nanoassembies. Notably, PPa solution was quickly cleared, accompanied by a lower tumor accumulation. Different from PPa, two nanoassembies exhibited more lightful fluorescence intensity in tumor tissues, especially PPa@GA/DSPE-PEG_2K_ NPs, consistent with the pharmacokinetic results.

**Figure 5.**
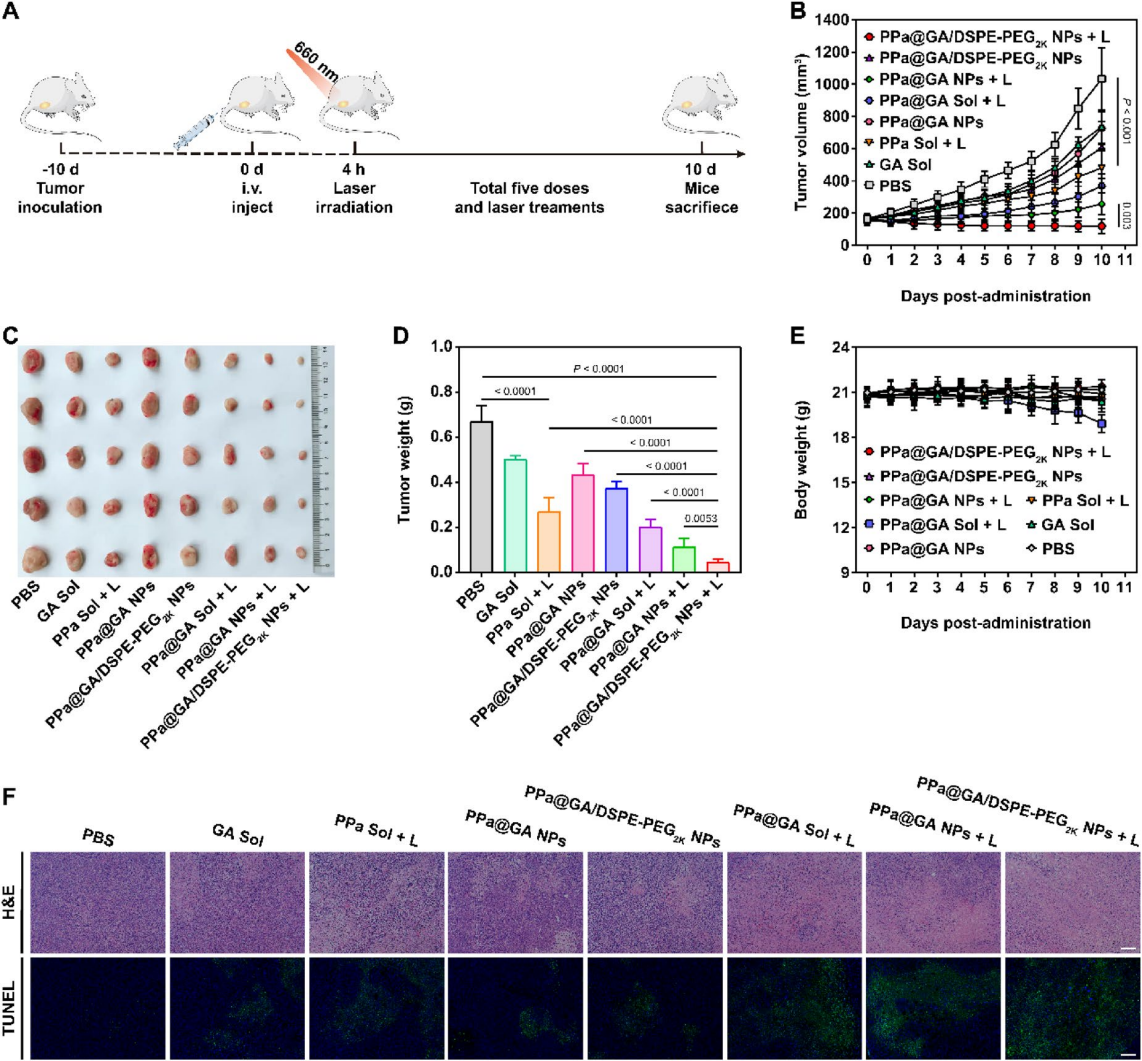
*In vivo* antitumor efficacy of dual-drug nanoassemblies against 4T1 xenograft tumors. A: Experimental design. B: Tumor growth profiles treated with different formulations. C: Images of the tumors collected from the mice after the last treatment. D: Tumor weights after the last treatment. E: Body weight changes. F: Images of the tumor sections examined by H&E and TUNEL. Scale bar, 100 μm.

### *In vivo* antitumor activity

The *in vivo* antitumor activity of the nanoassemblies was evaluated using 4T1 tumor-bearing mouse model (Figure 5(A)). As shown in Figure 5(B–D), the tumor volume of PBS-treated mice had grown to 1000 mm^3^ on day 10 of treatment. Compared to PBS group, GA solution, PPa@GA NPs, and PPa@GA/DSPE-PEG_2K_ NPs without laser exhibited moderate suppression on tumor growth. By contrast, the laser-irradiated combined treatment groups showed more effective tumor growth inhibition. As expected, PPa@GA/DSPE-PEG_2K_ NPs + L demonstrated superior antitumor activity to PPa solution + L, PPa@GA + L, and PPa@GA NPs + L, mainly due to several advantages including good colloidal stability, high cellular uptake, prolonged blood circulation time, high tumor accumulation, and the potential ability of GA and PPa to synergistically enhance ROS accumulation. Moreover, the results of H&E staining and TUNEL assay also demonstrated potent tumor destruction in laser-treated PPa@GA/DSPE-PEG_2K_ NPs group (Figure 5(F)).

Finally, the therapeutic safety of nanoassemblies was preliminarily investigated. During the treatment, the mice’s body weight was recorded daily and no significant loss was found (Figure 5(E)). Blood analysis of liver and kidney function revealed no abnormalities, and H&E sections of tissues and organs showed no significant injury (Supplemental Figures S6 and S7). These results proved that PPa@GA/DSPE-PEG_2K_ NPs was a safe and effective antitumor agent.

## Conclusion

In summary, we reported a dual-drug nanoassembly of PPa and GA to enhance PDT efficiency. PPa and GA were found to be easily co-assembled into stable NPs without any other excipients. An optimal formulation ratio (PPa/GA, 3:1) was obtained by screening for particle sizes of NPs and CIs of cytotoxicity. Moreover, to improve the stability of PPa@GA NPs, DSPE-PEG_2K_ (20%, wt%) was used to modify the nanoassembly in the preparation process. *In vitro* and *in vivo* evaluation, PPa@GA/DSPE-PEG_2K_ NPs demonstrated multiple advantages including high drug loading and colloid stability, high cellular uptake and cytotoxicity, increased blood circulation time and tumor accumulation as well as synergetic amplification of ROS in tumors. Ultimately, such advantages contributed to a potent antitumor activity against 4T1 tumor-bearing mice. Meanwhile, this rational design of drug pairing and efficient carrier-free delivery modality provides an excellent paradigm for enhanced PDT and drug combination therapy.

## Supplementary Material

Supplemental Material
