# Supplementary material for: Molecularly engineering a dual-drug nanoassembly for self-sensitized photodynamic therapy via thioredoxin impairment and glutathione depletion
Source: Drug Deliv. 2022 Nov 9;29(1):3281–90. doi: 10.1080/10717544.2022.2141920 (PMC9662020; doi:10.1080/10717544.2022.2141920)
Supplement: Supplemental Material [file IDRD_A_2141920_SM9468.docx]

**Supporting Information**

Title:

**Molecularly Engineering a Dual-Drug Nanoassembly for Self-Sensitized Photodynamic Therapy via Thioredoxin Impairment and** **Glutathione Depletion**

**Authors:** Hongyuan Zhang^1,a^, Zhiqiang Kong^1,a^, Ziyue Wang^1,a^, Yao Chen^1^, Shenwu Zhang^1*^, Cong Luo^1*^

**Affiliations:**

^1^Department of Pharmaceutics, Wuya College of Innovation, Shenyang Pharmaceutical University, Shenyang 110016, PR China

^a^These authors contributed equally to this work.

***Corresponding authors:**

Shenwu Zhang, Ph.D. and Cong Luo, Ph.D.

Department of Pharmaceutics, Wuya College of Innovation, Shenyang Pharmaceutical University, 103 Wenhua Road, Shenyang 110016, China

Tel: +86-024-23986321; Fax: +86-024-23986321

E-mail address: zhangshenwu@aliyun.com; luocong@syphu.edu.cn


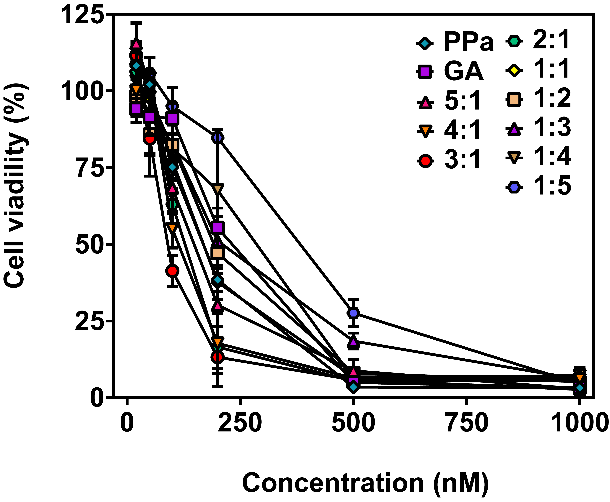


**Figure S1.** Screening the optimal synergy dose ratio of PPa and GA through cytotoxicity evaluation on 4T1 cells after treatment with different formulations laser irradiation after 4 h (660 nm, 50 mW/cm^2^, 5 min).


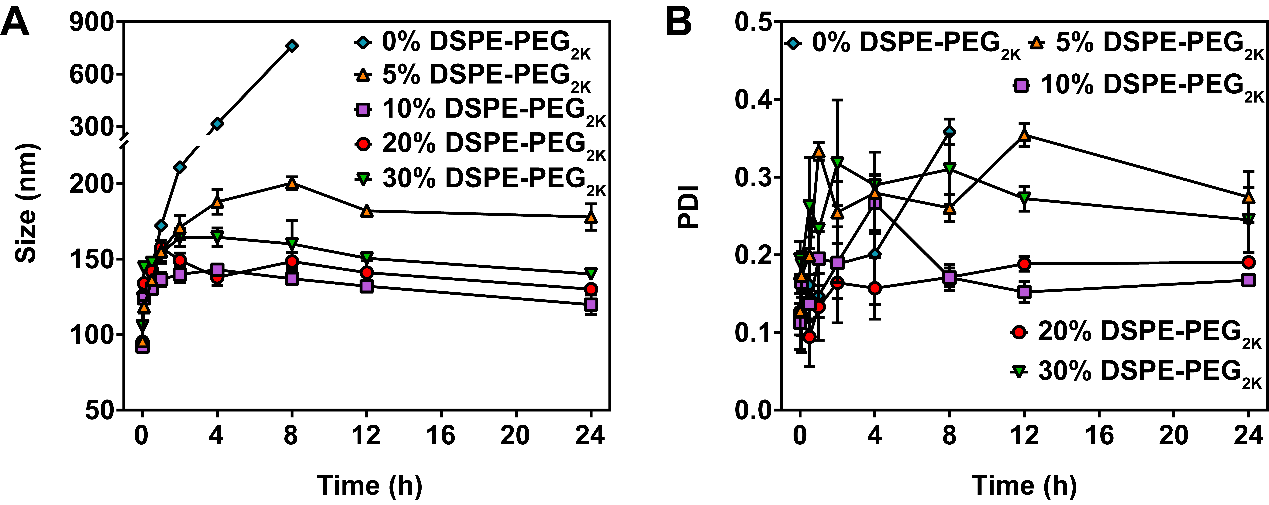


**Figure S2.** Screening PEG dosage of the formulation by comparing the stability of different formulations in PBS (pH 7.4).


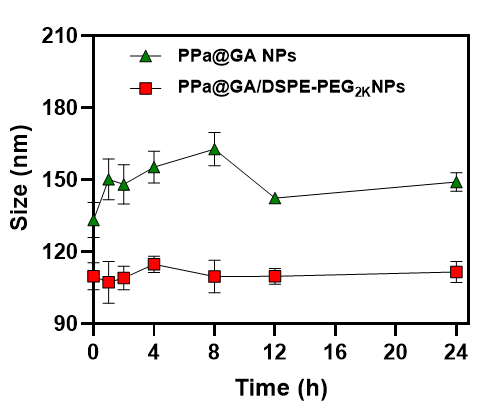


**Figure S3.** Colloidal stability of PPa@GA NPs and PPa@GA/DSPE-PEG_2K_ NPs in the PBS (pH 6.5).


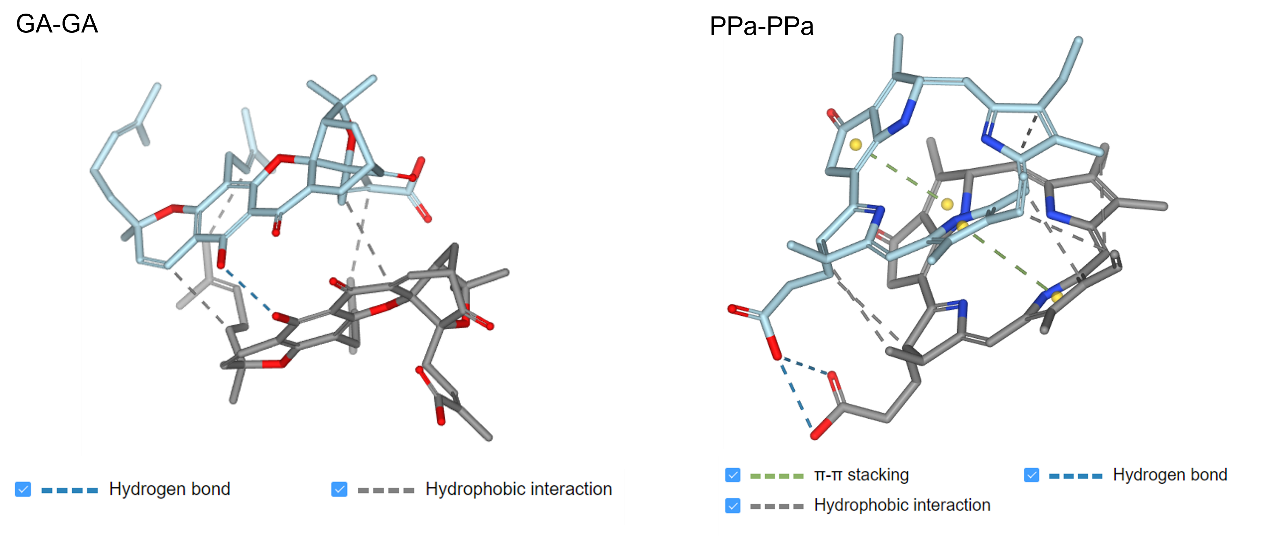


**Figure S4.** Molecular docking results of PPa-PPa and GA-GA.


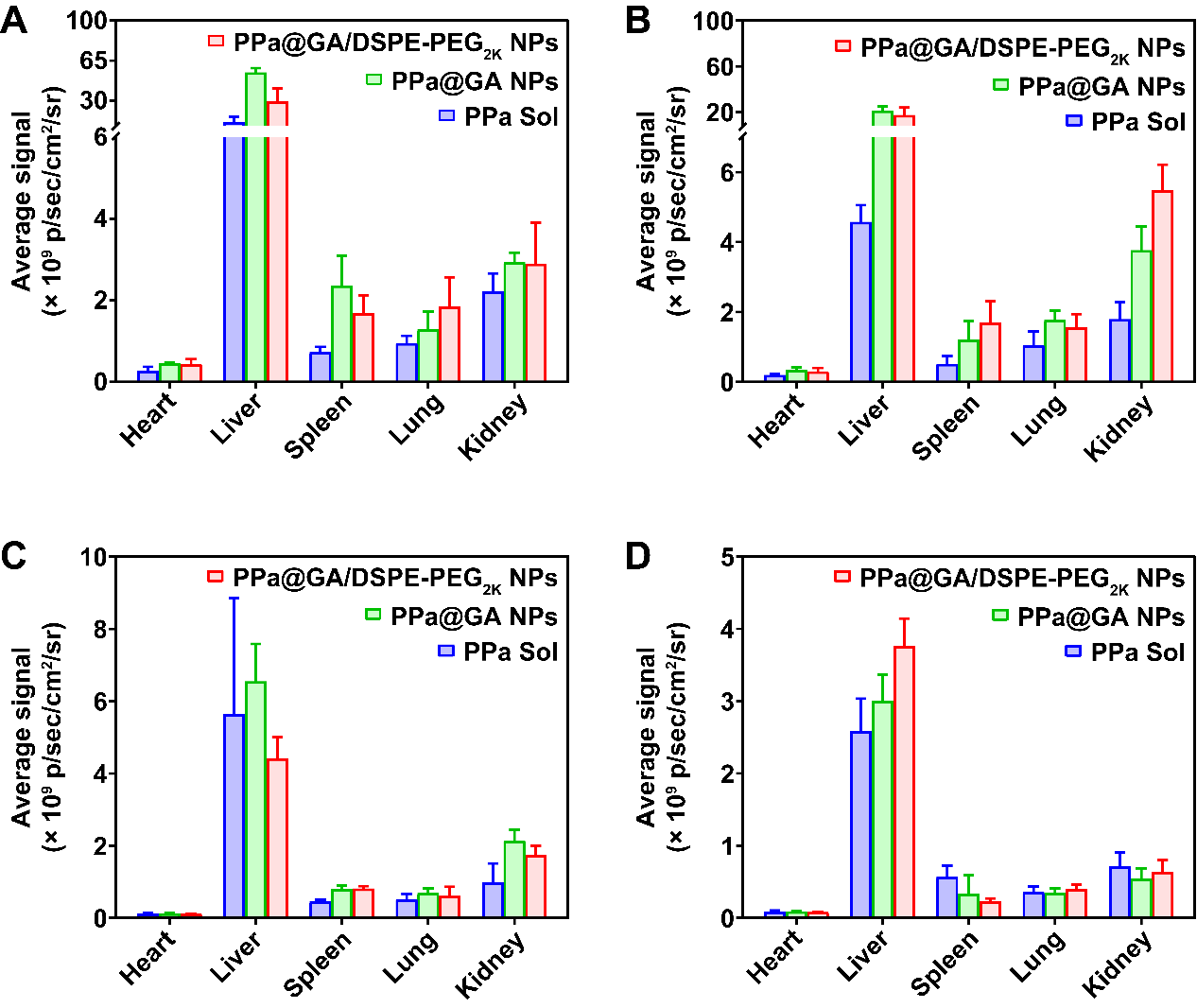


**Figure S5.** Quantitative analysis of *ex vivo* biodistribution in major organs at 2 h (A), 4 h (B), 8 h (C) and 12 h (D).


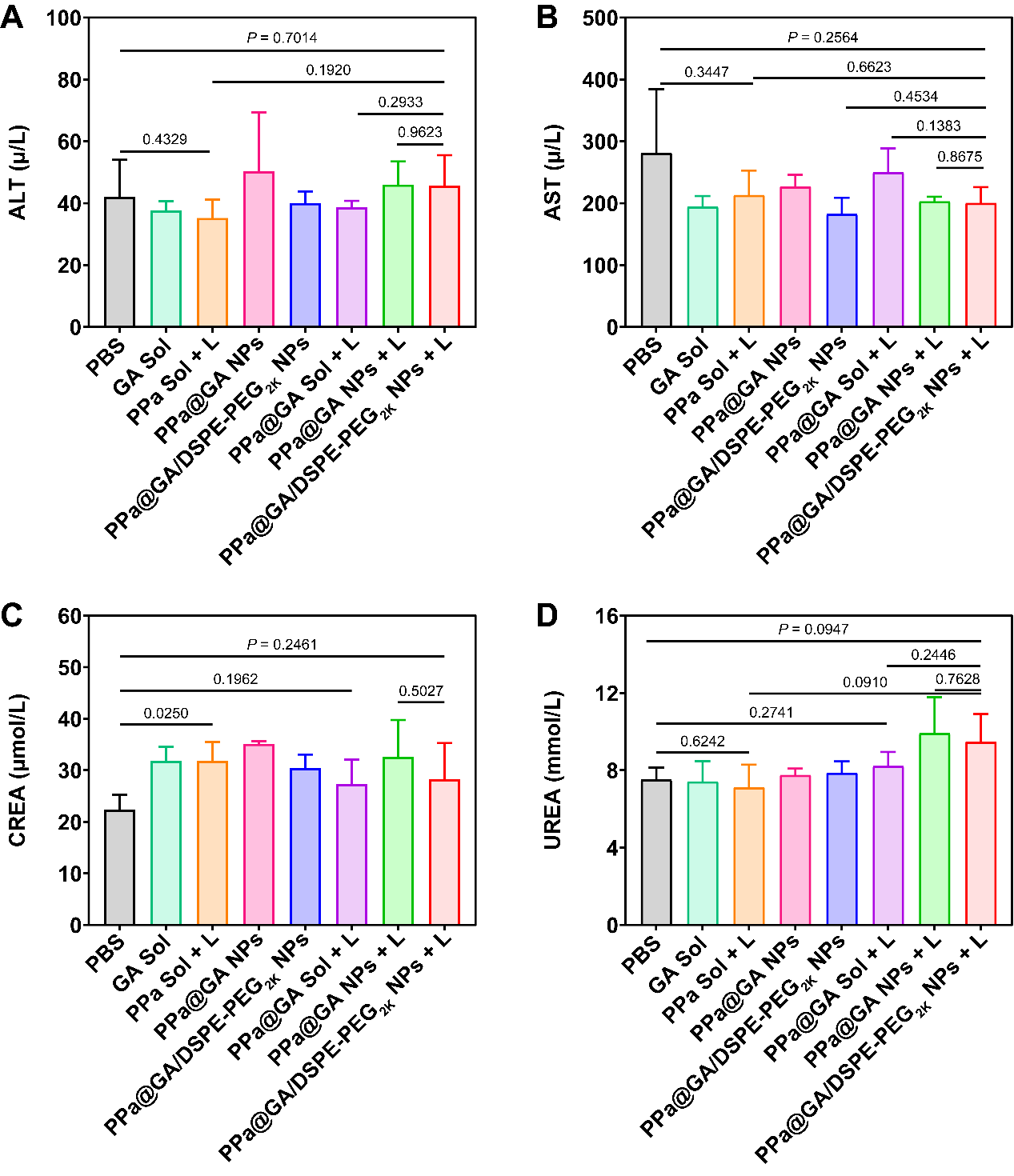


**Figure S6.** Hepatic and renal function indicators of 4T1 tumor-bearing mice after treatment (n=3). (A) ALT: alanine aminotransferase. (B) AST: aspartate aminotransferase. (C) CREA: creatinine. (D) UREA: urea nitrogen.


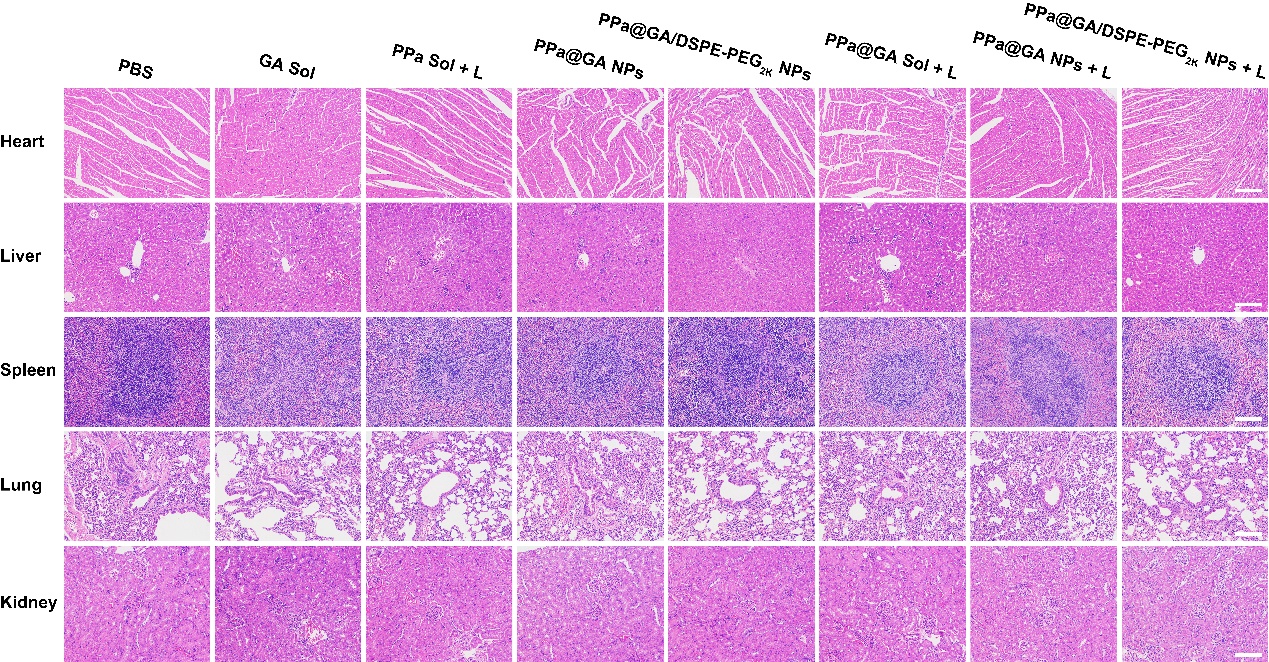


**Figure S7.** H&E staining images of heart, lung, spleen, lung and kidney after treatments. H&E staining: 200 × magnification. Scale bar, 100 μm.

**Table S1.** Screening the optimal synergy dose ratio of PPa and GA through cytotoxicity evaluation (n=3).

| PPa : GA | IC_50_ of 4T1 cells (nM) | CI in 4T1 cells |
| --- | --- | --- |
| 5 : 1 | 166.50 | 0.90 |
| 4 : 1 | 125.10 | 0.67 |
| 3 : 1 | 97.14 | 0.52 |
| 2 : 1 | 136.30 | 0.72 |
| 1 : 1 | 179.30 | 0.91 |
| 1 : 2 | 188.90 | 0.93 |
| 1 : 3 | 226.70 | 1.10 |
| 1 : 4 | 247.20 | 1.19 |
| 1 : 5 | 394.30 | 1.88 |

**Table S2.** Cytotoxicity (IC_50_ values) of PPa Sol and GA Sol (MTT assay).

| Formulations | IC_50_ of 4T1 cells (nM) |
| --- | --- |
| PPa Sol | 179.5 |
| GA Sol | 217.2 |

**Table S3.** Characterization of PPa/GA nanoassemblies at various molar ratios.

| PPa : GA | Size (nm) | PDI |
| --- | --- | --- |
| 5 : 1 | 136.2 ± 4.2 | 0.128 ± 0.030 |
| 4 : 1 | 152.1 ± 5.9 | 0.113 ± 0.024 |
| 3 : 1 | 162.2 ± 6.0 | 0.128 ± 0.044 |
| 2 : 1 | 145.4 ± 5.9 | 0.091 ± 0.071 |
| 1 : 1 | 163.3 ± 5.0 | 0.185 ± 0.044 |
| 1 : 2 | 185.2 ± 4.0 | 0.140 ± 0.043 |
| 1 : 3 | 195.2 ± 2.1 | 0.306 ± 0.049 |
| 1 : 4 | 225.2 ± 17.6 | 0.253 ± 0.046 |
| 1 : 5 | 222.1 ± 9.3 | 0.189 ± 0.076 |

**Table S4.** Characterization of PEGylated NPs and non-PEGylated NPs.

| Nanoassemblies | ^a)^Size (nm) | ^b)^PDI | ^c)^DL_PPa_ | ^c)^DL_GA_ | ^a)^Zeta (mV) |
| --- | --- | --- | --- | --- | --- |
| PPa@GA NPs | 126.9 ± 2.6 | 0.158 ± 0.048 | 71.8% | 28.2% | -21.4 ± 1.2 |
| PPa@GA/DSPE-PEG_2K_ NPs | 95.8 ± 2.2 | 0.120 ± 0.023 | 57.5% | 22.5% | -29.7 ± 3.4 |

a) Mean diameters and zeta potential of nanoassemblies were determined by DLS. b) Polydispersity index of micelles size. c) Drug-loading of PPa or GA was calculated by the molecular weight of drugs and the amount of DSPE-PEG_2K_.

**Table S5.** Molecular docking parameters of PPa-PPa, GA-GA and PPa-GA.

| Name | Affinity (kcal/mol) |
| --- | --- |
| GA-GA | -4.9 |
| PPa-PPa | -6.6 |
| PPa-GA | -6.0 |

**Table S6.** Cytotoxicity (IC_50_ values) of PPa Sol, GA Sol, PPa@GA Sol and hybrid nanoassemblies (MTT assay).

| Formulations | 4T1 (nM) | |
| --- | --- | --- |
|  | Laser (-) | Laser (+) |
| PPa Sol | 1944.0 | 157.7 |
| GA Sol | 379.0 | - |
| PPa@GA Sol | 530.3 | 98.9 |
| PPa@GA NPs | 586.0 | 83.5 |
| PPa@GA/DSPE-PEG_2K_ NPs | 771.6 | 49.3 |

**Table S7.** Pharmacokinetic parameters of PPa Sol, PPa@GA NPs and PPa@GA/DSPE-PEG_2K_ NPs (n = 6).

| Formulations | ^a)^C_max_ | ^b)^AUC_0-24h_ | ^c)^t_1/2_ | ^d)^MRT |
| --- | --- | --- | --- | --- |
| PPa Sol | 4.62 ± 1.18 | 11.07 ± 5.23 | 3.85 ± 1.00 | 4.16 ± 0.64 |
| PPa@GA NPs | 6.96 ± 1.35 | 32.46 ± 10.51 | 5.64 ± 1.14 | 5.80 ± 0.92 |
| PPa@GA/DSPE-PEG_2K_ NPs | 14.81 ± 1.62 | 65.12 ± 8.90 | 5.84 ± 0.63 | 7.01 ± 0.43 |

^a)^ The maximum plasma concentration (nmol/mL). ^b)^ Area under the plasma concentration-time curve to infinity (nmol·h/mL). ^c)^ Half-life (h). ^d)^ Mean residence time (h).
